# Thiotepa–fludarabine–treosulfan conditioning for 2nd allogeneic HCT from an alternative unrelated donor for patients with AML: a prospective multicenter phase II trial

**DOI:** 10.1038/s41409-022-01777-5

**Published:** 2022-08-18

**Authors:** Jürgen Finke, Claudia Schmoor, Matthias Stelljes, Andreas Burchert, Peter Dreger, Ute Hegenbart, Eva-Maria Wagner-Drouet, Martin Bornhäuser, Kristina Sohlbach, Natalie Schub, Christian Reicherts, Guido Kobbe, Bertram Glass, Hartmut Bertz, Olga Grishina

**Affiliations:** 1 Department of Hematology, Oncology and Stem Cell Transplantation, Faculty of Medicine and Medical Center—University of Freiburg, Freiburg, Germany; 2 Clinical Trials Unit, Faculty of Medicine and Medical Center—University of Freiburg, Freiburg, Germany; 3 Department of Medicine A, Hematology and Oncology, University of Muenster, Münster, Germany; 4 Department of Internal Medicine, Hematology, Oncology and Immunology, Philipps University Marburg and University Hospital Gießen and Marburg, Campus Marburg, Marburg, Germany; 5 Department of Medicine V, University of Heidelberg, Heidelberg, Germany; 6 Medical Department III, Hematology, Medical Oncology and Pneumology, University Mainz, Mainz, Germany; 7 Medical Department I, University Hospital Carl Gustav Carus, Technische Universitat Dresden, Dresden, Germany; 8 Division of Stem Cell Transplantation and Immunotherapy, 2nd Department of Medicine, University of Kiel, Kiel, Germany; 9 Medical Faculty, University Hospital Düsseldorf, Heinrich Heine University, Düsseldorf, Germany; 10 Department of Hematology, Oncology and Stem Cell Transplantation, Asklepios Klinik St Georg, Hamburg, Germany

**Keywords:** Phase II trials, Drug development

## Abstract

Therapeutic options for patients with AML relapsing after allogeneic HCT range from chemotherapy or hypomethylating agents with or without donor lymphocyte infusions to a 2nd allogeneic HCT. Available data are based on retrospective single center or registry studies. The aim of this multicenter trial was to investigate prospectively intensive conditioning with Thiotepa, Fludarabine and Treosulfan (TFT) for 2nd allogeneic HCT from an alternative unrelated donor in patients with AML relapse > 6 months after a 1st allogeneic HCT. Primary endpoint was disease-free survival (DFS) at one year after 2nd HCT. 50 patients median age 53.5 years, in CR/PR (34%) or active relapse (66%) were included. 33 of 38 patients (86.8%) with available data achieved CR 100 days post transplant. 23 patients were alive and free of relapse at primary endpoint one year after 2nd HCT (DFS rate 0.46, 95%-CI (0.32–0.61). Three-year rates of DFS, relapse, non-relapse mortality, and overall survival were 0.24, 95%-CI (0.13–0.36); 0.36 (0.25–0.52); 0.40 (0.29–0.57); and 0.24 (0.13–0.37). Second HCT with TFT conditioning is feasible and has high anti-leukemic efficacy in chemosensitive or refractory AML relapse after prior allogeneic HCT. Still, relapse rates and NRM after 2nd allogeneic HCT remain a challenge. The trial is registered in the German Clinical Trials Registry (number DRKS00005126).

## Introduction

Relapse of acute myeloid leukemia (AML) after allogeneic hematopoietic cell transplantation (HCT) has a dismal prognosis [1]. Apart from best supportive care, therapeutic options include treatment with drugs, cellular approaches like donor lymphocyte infusions (DLI) [2], combinations of both [3, 4], or another allogeneic transplantation [5]. In clinical practice, treatment is chosen depending on timing, availability and the condition of the patient, and frequently several treatment modalities are applied sequentially e.g. chemotherapy, DLI, and 2nd HCT [6]. Retrospective single or multicenter analyses as well as registry data indicate a 2-year survival probability of about 20% [1, 5, 7, 8]. Risk factors for outcome after 2nd HCT were relapse within 6 months after 1st transplantation, older age, and being not in remission prior to 2nd HCT. [5, 9–11] Considering a 2nd HCT, various factors possibly influencing the outcome could be addressed e.g. conditioning, GvHD prophylaxis, and donor choice. Apart from a different conditioning regimen, we therefore determined to use an alternative unrelated donor to possibly increase the graft versus leukaemia (GvL) effect by transplanting a novel immune system.

Relapse is the major cause of failure after 2nd HCT, followed by NRM in second place. Therefore, in a 2nd transplantation, adequate conditioning is a prerequisite to induce complete remission (CR) as well as to avoid toxicities. Different conditioning regimens have been used with prohibitive toxicities after standard full myeloablative conditioning, whereas increased relapse rates have been observed when using low dose conditioning. So far, no standard conditioning for this clinical situation has been established. Both treosulfan and thiotepa have shown activity as alkylating agents together with fludarabine for HCT conditioning, and combinations are in use in paediatric HCT [12–18].

Here we present the results of the first prospective multicenter trial for 2nd allogeneic HCT in patients with AML relapse after a 1st allogeneic HCT using peripheral blood derived hematopoietic cells from an alternative unrelated donor after a uniform double alkylator conditioning regimen.

## Methods

### Study design

This study was designed as an open-label, non-randomized, multicentre study to investigate efficacy and safety of a predefined conditioning regimen (TFT/thiotepa–fludarabine–treosulfan) in patients with AML relapsing from prior allogeneic HCT undergoing 2nd allogeneic PBSCT from an unrelated donor different from the donor at first transplantation. Eligible were patients aged 18–65 years with AML with sensitive or refractory hematologic relapse (≥20% blasts) later than 6 months after prior 1st allogeneic HCT, ECOG performance status of ≤2, adequate organ function, with an HLA-A, -B, -C and -DRB1 matched or mismatched (at maximum 2/8 alleles mismatched) unrelated donor. CR prior to 2nd HCT was no prerequisite.

### Procedures

All patients received myeloablative conditioning consisting of Thiotepa 3 × 5 mg/kg (day −8 to day −6), Fludarabine 3 × 30 mg/m^2^ (day −8 to day −6), Treosulfan 3 × 12 g/m^2^ (day −5 to day −3), and a GvHD prophylaxis with ATLG Grafalon (Neovii) 3 × 10 mg/kg (day −3 to day −1) and cyclosporine A (CyA) starting at day −1 and tapering between day +60 and +80, if no GvHD, and mycophenolic acid (MPA)/mycophenolate mofetil (MMF) starting day 0 until day +48.

Primary endpoint was disease-free survival (DFS) defined as being alive and free of disease at 1-year post 2nd HCT. Secondary endpoints were relapse, relapse mortality, non-relapse mortality (NRM), overall survival (OS), acute and chronic GvHD, engraftment, and adverse events (AEs).

The study was performed in nine German centers and was coordinated by the Clinical Trials Unit (CTU), Medical Center—University of Freiburg. Approvals of the German Federal competent authority (BfArM), as well as ethics committees, were obtained and patients gave written consent according to the Declaration of Helsinki. During the clinical trial, quality control was ensured through monitoring, auditing, and supervision by state authorities, if applicable. The CTU was responsible for project coordination, statistical planning, and analysis, data management, clinical monitoring, and pharmacovigilance.

### Statistical analysis

The study protocol including statistical planning was registered in advance of conducting the trial in the German Clinical Trials Registry (number DRKS00005126). Efficacy analyses were performed in the full analysis set (FAS) including all patients registered for the study, for whom the conditioning regimen TFT and the GvHD prophylaxis regimen CyA, MPA/MMF, ATLG has started, and for whom allogeneic PBSCT from an unrelated donor has been performed. Safety analyses of AEs, infections, and deaths were performed in the safety population including all patients who received at least one dose of the TFT regimen. Sample size calculation was based on the primary endpoint DFS. 50 patients were to be included to show at one-sided level *α* = 0.1 with a power of 90% that the probability of DFS at 1-year post 2nd HCT is >23%, when it is ≥40% in reality. The study was to be regarded as successful when ≥16 out of 50 patients are alive and free of disease after 1 year. The probability of DFS at 1-year post 2nd HCT was estimated as relative frequency with 80% (corresponding to the planned test procedure) and with 95%-CI (for reasons of comparability with reported results in the literature) based on the exact binomial distribution.

For the analysis of time-to-event variables over time, for which no competing events have to be considered, i.e. DFS time and OS time, the probability of an event over time was estimated by the Kaplan-Meier method with 95%. For the analysis of time-to-event variables, for which competing events have to be considered, e.g. time to relapse with NRM as competing event, time to NRM with relapse as competing event, time to acute GvHD or chronic GvHD with relapse and death as competing events, the probability of event over time was estimated by the Aalen–Johansen estimator with 95%-CI. Comparisons between patient groups were performed with Cox regression models, calculating two-sided Wald tests and hazard ratios (HRs) with two-sided 95%-CIs.

The effects of the following prognostic factors on DFS, relapse, NRM, and OS were analysed in univariate Cox regression models: patient age, donor age, patient and donor CMV status, time to relapse after 1st HCT, chronic GvHD after 1st HCT, cytogenetic risk [19], LDH before 2nd HCT, and remission status before 2nd HCT. The effects of the factors patient age (continuous), time to relapse after 1st HCT (continuous), and remission status before 2nd HCT (CR/PR vs. relapse/refractory) were additionally analysed in multiple Cox regression models. The study was not planned for a reliable analysis of the effects of prognostic factors. So, the results have to be interpreted in a descriptive sense.

AEs were coded with MedDRA and the incidences defined by preferred term were calculated as the number of patients who experienced at least one AE with the respective preferred term in percentage of the total number of patients in the safety population.

## Results

### Patients and treatment

Between March 2014 and March 2017, 52 patients from 9 German centers were enrolled and started the TFT conditioning regimen for the 2nd HCT (safety population). The FAS included 50 patients after exclusion of 2 patients due to death prior to 2nd HCT or transplantation from a related donor. One patient with a relapse 5.2 months after the 1st HCT entered the study. For patients’ baseline characteristics, disease status prior to and after 1st HCT, and details of 2nd HCT in the full analysis set, see Table 1. Median age was 53.5 years, 20% of the patients were older than 60 years. The HCT-CI score was ≥3 in 13 (26%) patients. Relevant findings concerning patients’ medical history were comorbidities in the pulmonary/respiratory system in 16 (32%) patients, and cardiovascular system in 14 (28%) patients. Median time between 1st HCT and relapse was 17.2 months. Of note, only 32% of patients received 2nd allogeneic HCT in CR. Eleven patients (22%) received a transplant with HLA-match less than 10/10.

**Table 1:** Patients’ baseline characteristics, disease status prior to and after 1st HCT, and details of 2nd HCT.

Characteristics	Value	Full analysis set (*n* = 50)
At enrolment		
Age (median, range)	Years	53.5 (19.0, 65.0)
Age (*n*, %)	18–40 years	10 (20%)
	41–50 years	13 (26%)
	51–60 years	17 (34%)
	>60 years	10 (20%)
Gender (*n*, %)	Male	32 (64%)
ECOG (*n*, %)	0	12 (24%)
	1	34 (68%)
	2	4 (8%)
HCT-CI (*n*, %)	0	20 (40%)
	1–2	17 (34%)
	≥3	13 (26%)
Before and after 1st HCT		
Remission status prior to 1st HCT (*n*, %)	CR	37 (74%)
	Relapse	2 (4%)
	Primary refractory	2 (4%)
	Other	9 (18%)
Cytogenetic risk [19] prior to 1st HCT (*n*, %)	Favorable	6 (12.5%)
	Intermediate I	14 (29.2%)
	Intermediate II	14 (29.2%)
	Adverse	14 (29.2%)
	Unknown	2
1st HCT: conditioning (*n*, %)	Myeloablative^a^	23 (46%)
1st HCT: donor (*n*, %)	Unrelated	39 (78%)
1st HCT: stem cell source (*n*, %)	Peripheral blood	48 (96%)
Acute GvHD after 1st HCT (*n*, %)	I–IV	17 (34%)
	III–IV	5 (10%)
Chronic GvHD after 1st HCT (*n*, %)	Yes	20 (40%)
Time to relapse after 1st HCT (median, range)	Months	17.2 (5.2, 65.6)
Time to relapse after 1st HCT (*n*, %)	≤12 months	20 (40%)
	>12–24 months	12 (24%)
	>24 months	18 (36%)
Relapse therapy (*n*, %)	Induction CT	36 (72%)
	1 cycle	22
	2 cycles	5
	3–6 cycles	4
	Azacytidine/Decitabine	11 (22%)
	DLI	11 (22%)
2nd HCT		
Relapse status prior to 2nd HCT (*n*, %)	CR	16 (32%)
	PR	1 (2%)
	Relapse	33 (66%)
Time from AML diagnosis to 2nd HCT (median, range)	Months	27.9 (0.7, 103.1)
Time from 1st HCT to 2nd HCT (median, range)	Months	20.6 (7.1, 97.3)
Time from relapse after 1st HCT to 2nd HCT (median, range)	Months	2.5 (0.5, 64.9)
2nd HCT: donor age (median, range)	Years	30 (18, 54)
2nd HCT: patient/donor sex (*n*, %)	Male/Female	5 (10%)
2nd HCT: patient/donor CMV (*n*, %)	Both negative	17 (34%)
2nd HCT: HLA-match (*n*, %)	Match (8/8)—4 digit	39 (78%)
	Match (10/10) incl. DQB1—4 digit	39 (78%)
	Mismatch	11 (22%)
2nd HCT: number of CD34 + cells (median, range)	×10^6^/kg	8.0 (2.9, 15.8)

^a^Myeloablative: as indicated by centers' investigators including BU/CY and TBI-containing regimen.

### Primary endpoint

With regard to the primary endpoint, 23 out of 50 patients in the full analysis set were alive and free of relapse at one year after 2nd HCT (DFS rate 0.46, 95%-CI (0.32–0.61). The two-sided 80%-CI of the DFS rate was (0.36–0.56). It could therefore be shown at one-sided *α* = 0.1 that the probability of DFS at 1-year post 2nd HCT is higher than 0.36. Thus, the success criterion for the primary endpoint was fulfilled. Of the remaining patients, 4 patients were alive after relapse, 9 patients died after relapse, and 14 patients died because of other reasons (infection *n* = 6, infection after acute GvHD *n* = 6, PTLD *n* = 2), at 1 year after 2nd HCT.

### Secondary endpoints

The rates of engraftment with respect to ANC and platelets within 3 months were as follows: ANC > 0.5/nL: 0.94, 95%-CI (0.87–1.00), ANC > 1.0/nL: 0.92, 95%-CI (0.85–1.00), platelets >20/nL: 0.80, 95% CI (0.70–0.92), with 4 patients dying from infections before day+30. Median time to ANC > 0.5/nl and 1.0/nL was 15 and 18 days, respectively, and median time to platelets >20/nL was 17 days.

At day +100 post-transplant, bone marrow diagnostics were performed in 38 of 39 patients alive at this time, demonstrating morphologic CR in 33 (86.8%) of them. Complete donor chimerism was demonstrated for 34 (94.4%) out of 36 patients with available data.

The one-year incidence rates for acute grade °II–IV, grade °III–IV and moderate/severe chronic GvHD were 0.42, 95%-CI (0.30–0.58); 0.26, 95%-CI (0.16–0.42); and 0.22, 95%-CI (0.13–0.37), respectively.

Median follow-up time for relapse and death was 50.6 months. 20 patients experienced a relapse (17 of them died after relapse), and 20 patients died from other reasons (NRM). Reasons of NRM were infection with (*n* = 7) or without (*n* = 6) GvHD, EBV-PTLD (*n* = 2), secondary malignancy (*n* = 1), sudden cardiac death (*n* = 1) and unknown (*n* = 3).

**Fig. 1:**
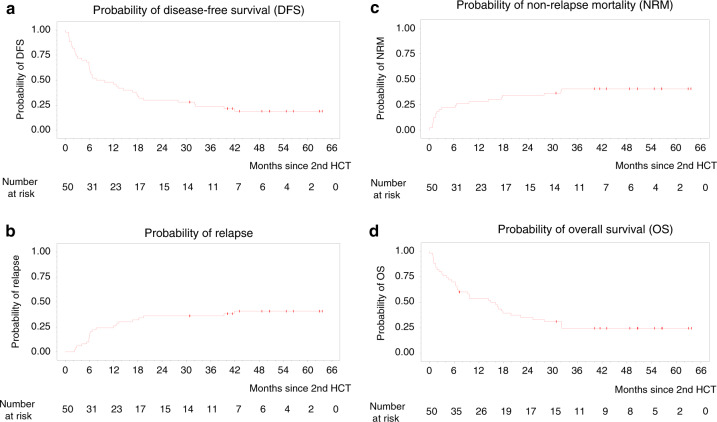
Results of relapse and death over time. **a** Probability of disease-free survival (DFS). **b** Probability of relapse. **c** Probability of non-relapse mortality (NRM). **d** Probability of overall survival (OS).

The estimated probabilities of DFS, relapse, NRM, and OS over time are shown in Fig. 1a–d. At 1-year post 2nd HCT, the DFS rate was 0.46, 95%-CI (0.32–0.59), the relapse rate was 0.26, 95%-CI (0.16–0.42), the NRM rate was 0.28, 95%-CI (0.18–0.44), and the OS rate was 0.54, 95%-CI (0.39–0.66). At three years post 2nd HCT, the DFS rate was 0.24, 95%-CI (0.13–0.36), the relapse rate was 0.36, 95%-CI (0.25–0.52), the NRM rate was 0.40, 95%-CI (0.29–0.57), and the OS rate was 0.24, 95%-CI (0.13–0.37).

**Fig. 2:**
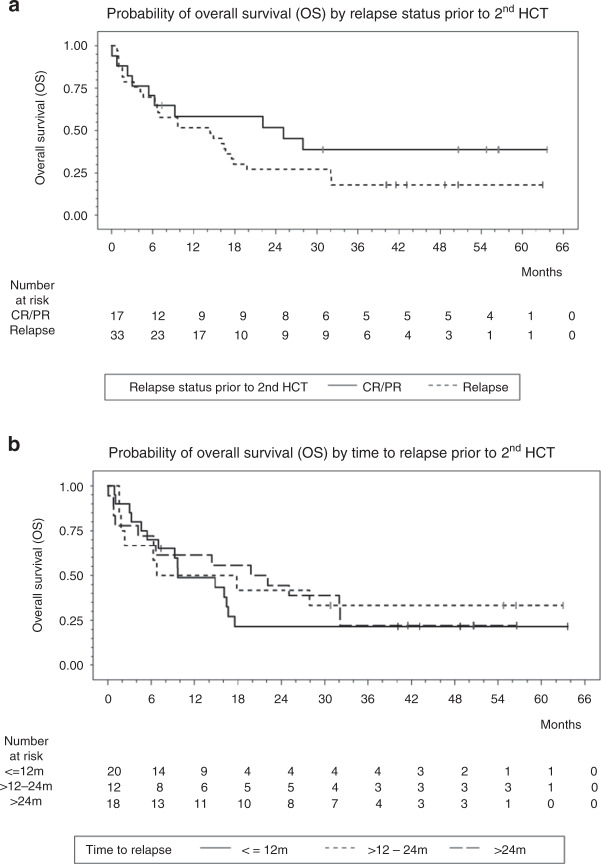
Overall survival by patient and disease characterisitics. **a** Probability of overall survival (OS) by relapse status prior to 2nd HCT. **b** Probability of overall survival (OS) by time to relapse prior to 2nd HCT.

No significant effects of prognostic factors on relapse and death were observed (see Supplement Tables S1–S4). In general, it has to be considered that the study was not planned for a reliable analysis of the effects of prognostic factors. The numbers of observed events are small, and 95%-CI of estimated hazard ratios are large. The OS rates by relapse status prior to 2nd HCT and time to relapse between 1st HCT and relapse are shown in Fig. 2.

At follow-up month 12, 7 of 24 (29.2%) patients with available data had an ECOG grade 0, 16 (66.7%) patients grade 1, and 1 (4.2%) patient grade 2.

Adverse events of CTCAE grade ≥3 were observed in all 52 patients in the safety population with sepsis/septic shock in 16 (see Supplement Table S5). Non-haematologic toxicities being at least severe (CTCAE grade ≥3) included mucosal inflammation in 22, pneumonia and nausea each in 9 patients, hyperbilirubinemia in 6, and VOD/SOS in 2 patients. 15 patients died due to infection with or without acute GvHD, among them 10 patients who had received intensive antileukemic chemotherapy for relapse prior to 2nd HCT, and severe encephalopathy occurred in 5 of these patients with a medical history of extensive treatment including intrathecal and high dose blood-brain barrier penetrating chemotherapy, and cranial irradiation (1 patient).

## Discussion

In this prospective multicenter trial, a high rate of CR was achieved despite the fact that 66% of patients were not in CR prior to 2nd allogeneic HCT. The use of the 2 alkylating agents thiotepa and treosulfan with high myeloablative and immunosuppressive potency in combination with fludarabine for 2nd allogeneic transplantation appears feasible. The success criterion for the primary endpoint was fulfilled with the probability of DFS at 1 year higher than 0.36.

The toxicities observed are certainly of concern although not totally unexpected in this cohort of intensely pretreated patients. For example, the CNS toxicity observed in patients with prior CNS-directed treatments because of AML relapse in the CNS was possibly aggravated by the conditioning drugs fludarabine and thiotepa known to penetrate the blood-brain barrier. It could be speculated on the contributing effects of pretransplant chemotherapy since in our study, patients who had died due to infections had received intensive antileukemic chemotherapy for relapse prior 2nd allogeneic transplantation.

Recently, a transplant conditioning intensity (TCI) score based on early toxicities has been established from the EBMT database allowing the comparison of different conditioning regimens used in first allogeneic HCT as a continuous variable [20]. The conditioning regimen presented here used for 2nd allogeneic HCT with Thiotepa, Fludarabine, and Treosulfan is to be positioned in the upper part of the intermediate intensity range with a TCI score of 3.5. The TFT combination had been used mainly for non-malignant diseases in paediatric patients and with variations in dosing, resulting in transient skin toxicities with more intensive dosing [21, 22]. No formal trial with the TFT conditioning has been published for adult patients. A retrospective study compared Fludarabine-Treosulfan to Thiotepa-Busulfan-Fludarabine in adult patients with refractory or relapsed AML demonstrating similar outcome [23]. Treosulfan with a dose of 3 × 10 g/m^2^ plus Fludarabine resulted in better survival in a randomised trial compared to Fludarabine plus Busulfan 2 days [18]. Recently, Treosulfan 3 × 10–14 g/m^2^, together with Fludarabine plus/minus Thiotepa 2 × 5 mg/kg has been licensed in Europe for conditioning for allogeneic HCT in adults and children/adolescents with malignant and nonmalignant diseases. For the wider application in adult patients with an increasing age and also for 1st allogeneic HCT, in Freiburg we have adapted the TFT regimen accordingly with Thiotepa 2 × 5 mg/kg, Fludarabine 3 × 30 mg/m^2^, and Treosulfan 3 × 30 g/m^2^ and early results regarding, engraftment, toxicities, and CR rates are promising (JF unpublished data).

Our decision to change the donor for 2nd allogeneic transplantation was based on theoretical considerations for a different immune system. This is supported by one multicenter retrospective analysis finding a benefit [8], but not in other publications [5, 9, 24, 25]. Several immune-escape mechanism have been described in AML relapse, among other loss of HLA in mismatched transplantation, further supporting a change of donor [26]. The acute leukemia working party of the EBMT compared two groups of patients either receiving a 2nd allogeneic HCT from an unrelated (different donor in 79%) or a haploidentical (different in 93%) donor [25]. The 2 year survival was similar for both groups with around 30%, and early relapse, age and active disease were risk factors for OS [25]. Comparing the time periods 2000–2004 to 2015–2018, the 2-year survival after 2nd allogeneic HCT has improved in recent years for patients aged 18–50 years from 22.6% to 32.4% [27].

For the interpretation of that data it has to be kept in mind that frequently various methods of T cell depletion had been used for 1st allogeneic transplantation and were avoided subsequently for the 2nd transplantation. Similarly, when bone marrow was transplanted previously, the use of G-CSF mobilized peripheral blood-derived grafts from the same donor for the 2nd transplantation is likely to confer a better GvL effect [28]. Donor options have increased in recent years with the use of post-transplant cyclophosphamide as GvHD prophylaxis after matched or haplotransplantion [24, 25]. Based on current knowledge, the preference of the more rapidly available donor makes sense, when options also include mismatched or haploidentical related or mismatched unrelated donors. This strategy may help to avoid excessive treatments for relapse and hopefully results in patients entering 2nd transplantation in better condition. Choice of donor and GvHD prophylaxis are closely connected and both determine immune effects after allogeneic HCT. Based on randomised trials for 1st allogeneic transplantation, demonstrating the reduction of GvHD without increasing relapse risk [29–31], we decided to use ATLG in a reduced dose, compared to the randomised trial in 1st HCT [32], together with CSA and MMF/MPA to avoid toxicities from methotrexate and the risk of severe GvHD when transplanting PBSC from unrelated donors. This can be debated, especially with the availability of novel prophylaxis regimen using abatacept or PT-CY [33, 34]. Whatever will allow rapid tapering of immunosuppressive agents and the establishment of a GvL effect in time is warranted.

We did not observe any large effects of patient and disease factors on outcome. The results have to be interpreted within the context of patient inclusion criteria (only adults aged up to 65 years, relapse beyond 6 months after 1st transplantation) as well as the limits of patient numbers with 50 patients included in the trial. The study was not planned for a reliable analysis of the effects of prognostic factors. For AML relapse after 1st HCT the importance of a cellular therapy, DLI or 2nd allogeneic HCT, has been demonstrated repeatedly [1, 7]. However, DLI shows little effect in hematologic relapse [2, 7, 35]. Since outcome was not dependent on state of remission prior 2nd transplant in our trial, we favour a rapid proceeding to transplant once a donor is available and use the time interval for rather debulking and not enforcing CR by heavy treatment.

With a median follow up of 5.6 years, the 3-year probability of OS is 0.24 (95%-CI 0.13–0.37) after 2nd allogeneic transplantation for patients with AML relapsing after a 1st transplantation. This outcome is certainly sobering although indicating a fair chance for cure in this dismal situation. The 3-year survival rate of 0.24 compares favourably to a retrospective case series from Seattle where the majority of patients received a 2nd allogeneic HCT from a different donor after various reduced intensity conditioning regimens [36]. Late relapse and patient fitness were favourable prognostic factors, but not remission. Less NRM was observed after treosulfan conditioning and use of a different donor was not detrimental [36]. Although any direct comparison with published case series and retrospective registry cohorts may be biased, the strategy of a 2nd allogeneic hematopoietic cell transplantation is valid. However, despite advances in recent years, outcome clearly has to be further improved [27, 37].

The conditioning regimen presented here may be a good platform for this approach, even more since other conditioning regimens are more frequently in use for 1^st^ allogeneic transplantation thus allowing a change of drugs. Furthermore, our trial is the first using the combination of Thiotepa–Fludarabine–Treosulfan (TFT) for conditioning in allogeneic HCT for adult patients.

In conclusion, this first prospective trial on 2nd allogeneic HCT in patients with chemosensitive or refractory AML having failed a prior allogeneic HCT shows that TFT conditioning is feasible and has high anti-leukemic efficacy, resulting in long-term survival in one out of four patients in this poor-risk population. Although NRM deserves attention, these results may establish TFT as a platform in this indication and warrant exploration of this regimen in other transplantation settings for adult patients.

## Supplementary information

Supplement

## Data Availability

Original data are available at the Clinical Trials Unit (CTU), Medical Center—University of Freiburg.
