# Supplementary material for: Thiotepa–fludarabine–treosulfan conditioning for 2nd allogeneic HCT from an alternative unrelated donor for patients with AML: a prospective multicenter phase II trial
Source: Bone Marrow Transplant. 2022 Aug 18;57(11):1664–70. doi: 10.1038/s41409-022-01777-5 (PMC9630110; doi:10.1038/s41409-022-01777-5)
Supplement: Supplementary file 1 — Supplement [file 41409_2022_1777_MOESM1_ESM.docx]

**Supplement:**

| Table S1a: Analysis of effects of prognostic factors on disease-free survival - Univariate analyses |
| --- |

| *Factor* | *Value** | *Hazard ratio* | *95%-CI Lower limit* | *95%-CI Upper limit* | *p-value* |
| --- | --- | --- | --- | --- | --- |
| Patient age (continuous) | per 10 years | 0.994 | 0.769 | 1.285 | 0.9636 |
| Patient age | <=40 vs. >60 | 1.450 | 0.558 | 3.769 | 0.8174 |
| Patient age | 41-50 vs. >60 | 1.154 | 0.453 | 2.936 | - |
| Patient age | 51-60 vs. >60 | 1.451 | 0.600 | 3.513 | - |
| Time to relapse after 1st HCT (continuous) | per year | 0.983 | 0.772 | 1.253 | 0.8926 |
| Time to relapse after 1st HCT | <=12m vs. >24m | 1.104 | 0.548 | 2.223 | 0.7138 |
| Time to relapse after 1st HCT | >12-24m vs. >24m | 0.773 | 0.330 | 1.810 | - |
| chronic GvHD after 1st HCT | no vs. yes | 0.790 | 0.421 | 1.479 | 0.4607 |
| Cytogenetic risk | Favorable vs. Adverse | 1.498 | 0.517 | 4.342 | 0.7136 |
| Cytogenetic risk | Intermediate-I vs. Adverse | 1.280 | 0.563 | 2.910 | - |
| Cytogenetic risk | Intermediate-II vs. Adverse | 0.880 | 0.381 | 2.034 | - |
| Donor age (continuous) | per 10 years | 0.888 | 0.622 | 1.268 | 0.5146 |
| CMV status pat/don | P neg / D neg vs. other | 0.818 | 0.426 | 1.571 | 0.5462 |
| LDH (continuous) | per 100U/l | 0.977 | 0.828 | 1.154 | 0.7867 |
| LDH | <300U/l vs. >=300U/l | 0.994 | 0.437 | 2.260 | 0.9878 |
| Relapse status prior to 2nd HCT | CR/PR vs. Relapse | 0.781 | 0.402 | 1.519 | 0.4670 |

* In x vs. y notation, y is reference group.

|  |
| --- |
| Table S1b: Analysis of effects of prognostic factors on disease-free survival - Multiple Cox regression |

| *Factor* | *Value* | *Hazard ratio* | *95%-CI Lower limit* | *95%-CI Upper limit* | *p-value* |
| --- | --- | --- | --- | --- | --- |
| Patient age (continuous) | per 10 years | 0.942 | 0.708 | 1.253 | 0.6810 |
| Time to relapse after 1st HCT (continuous) | per year | 0.969 | 0.760 | 1.236 | 0.8005 |
| Relapse status prior to 2nd HCT | CR/PR vs. Relapse | 0.733 | 0.354 | 1.516 | 0.4015 |

|  |
| --- |
| Table S2a: Analysis of effects of prognostic factors on relapse - Univariate analyses |

| *Factor* | *Value* | *Hazard ratio* | *95%-CI Lower limit* | *95%-CI Upper limit* | *p-value* |
| --- | --- | --- | --- | --- | --- |
| Patient age (continuous) | per 10 years | 0.773 | 0.552 | 1.082 | 0.1340 |
| Patient age | <=40 vs. >60 | 3.627 | 0.958 | 13.728 | 0.1143 |
| Patient age | 41-50 vs. >60 | 0.935 | 0.187 | 4.673 | - |
| Patient age | 51-60 vs. >60 | 1.952 | 0.486 | 7.835 | - |
| Time to relapse after 1st HCT (continuous) | per year | 0.716 | 0.464 | 1.102 | 0.1291 |
| Time to relapse after 1st HCT | <=12m vs. >24m | 1.841 | 0.707 | 4.795 | 0.1430 |
| Time to relapse after 1st HCT | >12-24m vs. >24m | 0.462 | 0.096 | 2.229 | - |
| chronic GvHD after 1st HCT | no vs. yes | 0.794 | 0.325 | 1.937 | 0.6115 |
| Cytogenetic risk | Favorable vs. Adverse | 1.706 | 0.421 | 6.920 | 0.5365 |
| Cytogenetic risk | Intermediate-I vs. Adverse | 1.222 | 0.392 | 3.811 | - |
| Cytogenetic risk | Intermediate-II vs. Adverse | 0.585 | 0.165 | 2.077 | - |
| Donor age (continuous) | per 10 years | 0.839 | 0.511 | 1.379 | 0.4891 |
| CMV status pat/don | P neg / D neg vs. other | 0.948 | 0.386 | 2.329 | 0.9077 |
| LDH (continuous) | per 100U/l | 1.011 | 0.815 | 1.255 | 0.9183 |
| LDH | <300U/l vs. >=300U/l | 0.613 | 0.220 | 1.711 | 0.3501 |
| Relapse status prior to 2nd HCT | CR/PR vs. Relapse | 1.047 | 0.426 | 2.572 | 0.9206 |

|  |
| --- |
| Table S2b: Analysis of effects of prognostic factors on relapse - Multiple Cox regression |

| *Factor* | *Value* | *Hazard ratio* | *95%-CI Lower limit* | *95%-CI Upper limit* | *p-value* |
| --- | --- | --- | --- | --- | --- |
| Patient age (continuous) | per 10 years | 0.683 | 0.467 | 0.999 | 0.0495 |
| Time to relapse after 1st HCT (continuous) | per year | 0.676 | 0.440 | 1.040 | 0.0749 |
| Relapse status prior to 2nd HCT | CR/PR vs. Relapse | 0.739 | 0.281 | 1.942 | 0.5391 |

|  |
| --- |
| Table S3a: Analysis of effects of prognostic factors on non-relapse mortality - Univariate analyses |

| *Factor* | *Value* | *Hazard ratio* | *95%-CI Lower limit* | *95%-CI Upper limit* | *p-value* |
| --- | --- | --- | --- | --- | --- |
| Patient age (continuous) | per 10 years | 1.368 | 0.896 | 2.087 | 0.1463 |
| Patient age | <=40 vs. >60 | 0.243 | 0.028 | 2.089 | 0.4888 |
| Patient age | 41-50 vs. >60 | 1.262 | 0.399 | 3.989 | - |
| Patient age | 51-60 vs. >60 | 1.140 | 0.360 | 3.605 | - |
| Time to relapse after 1st HCT (continuous) | per year | 1.235 | 0.911 | 1.673 | 0.1732 |
| Time to relapse after 1st HCT | <=12m vs. >24m | 0.577 | 0.192 | 1.738 | 0.5807 |
| Time to relapse after 1st HCT | >12-24m vs. >24m | 0.982 | 0.348 | 2.769 | - |
| chronic GvHD after 1st HCT | no vs. yes | 0.786 | 0.325 | 1.900 | 0.5925 |
| Cytogenetic risk | Favorable vs. Adverse | 1.278 | 0.247 | 6.629 | 0.9661 |
| Cytogenetic risk | Intermediate-I vs. Adverse | 1.355 | 0.412 | 4.456 | - |
| Cytogenetic risk | Intermediate-II vs. Adverse | 1.239 | 0.392 | 3.922 | - |
| Donor age (continuous) | per 10 years | 0.945 | 0.567 | 1.577 | 0.8298 |
| CMV status pat/don | P neg / D neg vs. other | 0.697 | 0.267 | 1.819 | 0.4604 |
| LDH (continuous) | per 100U/l | 0.937 | 0.721 | 1.218 | 0.6252 |
| LDH | <300U/l vs. >=300U/l | 1.938 | 0.448 | 8.375 | 0.3756 |
| Relapse status prior to 2nd HCT | CR/PR vs. Relapse | 0.560 | 0.203 | 1.545 | 0.2628 |

|  |
| --- |
| Table S3b: Analysis of effects of prognostic factors on non-relapse mortality - Multiple Cox regression |

| *Factor* | *Value* | *Hazard ratio* | *95%-CI Lower limit* | *95%-CI Upper limit* | *p-value* |
| --- | --- | --- | --- | --- | --- |
| Patient age (continuous) | per 10 years | 1.378 | 0.870 | 2.182 | 0.1714 |
| Time to relapse after 1st HCT (continuous) | per year | 1.276 | 0.918 | 1.772 | 0.1465 |
| Relapse status prior to 2nd HCT | CR/PR vs. Relapse | 0.827 | 0.271 | 2.523 | 0.7382 |

|  |
| --- |
| Table S4a: Analysis of effects of prognostic factors on overall survival - Univariate analyses |

| *Factor* | *Value* | *Hazard ratio* | *95%-CI Lower limit* | *95%-CI Upper limit* | *p-value* |
| --- | --- | --- | --- | --- | --- |
| Patient age (continuous) | per 10 years | 1.135 | 0.862 | 1.494 | 0.3657 |
| Patient age | <=40 vs. >60 | 0.928 | 0.336 | 2.561 | 0.8119 |
| Patient age | 41-50 vs. >60 | 0.981 | 0.377 | 2.551 | - |
| Patient age | 51-60 vs. >60 | 1.356 | 0.561 | 3.280 | - |
| Time to relapse after 1st HCT (continuous) | per year | 0.991 | 0.772 | 1.271 | 0.9436 |
| Time to relapse after 1st HCT | <=12m vs. >24m | 1.160 | 0.555 | 2.427 | 0.8643 |
| Time to relapse after 1st HCT | >12-24m vs. >24m | 0.931 | 0.390 | 2.222 | - |
| chronic GvHD after 1st HCT | no vs. yes | 0.764 | 0.399 | 1.462 | 0.4163 |
| Cytogenetic risk | Favorable vs. Adverse | 1.213 | 0.383 | 3.838 | 0.8122 |
| Cytogenetic risk | Intermediate-I vs. Adverse | 1.143 | 0.503 | 2.597 | - |
| Cytogenetic risk | Intermediate-II vs. Adverse | 0.782 | 0.331 | 1.845 | - |
| Donor age (continuous) | per 10 years | 0.861 | 0.586 | 1.265 | 0.4453 |
| CMV status pat/don | P neg / D neg vs. other | 0.738 | 0.370 | 1.472 | 0.3879 |
| LDH (continuous) | per 100U/l | 0.938 | 0.773 | 1.137 | 0.5122 |
| LDH | <300U/l vs. >=300U/l | 1.113 | 0.464 | 2.672 | 0.8098 |
| Relapse status prior to 2nd HCT | CR/PR vs. Relapse | 0.637 | 0.308 | 1.321 | 0.2255 |

|  |
| --- |
| Table S4b: Analysis of effects of prognostic factors on overall survival - Multiple Cox regression |

| *Factor* | *Value* | *Hazard ratio* | *95%-CI Lower limit* | *95%-CI Upper limit* | *p-value* |
| --- | --- | --- | --- | --- | --- |
| Patient age (continuous) | per 10 years | 1.078 | 0.802 | 1.450 | 0.6189 |
| Time to relapse after 1st HCT (continuous) | per year | 0.996 | 0.775 | 1.281 | 0.9781 |
| Relapse status prior to 2nd HCT | CR/PR vs. Relapse | 0.683 | 0.314 | 1.484 | 0.3352 |

Table S5: Incidence of non-hematologic adverse events being at least severe (CTCAE grade 3-5) until death / end of follow-up

|  | Incidence*** | | 95% Confidence interval | |
| --- | --- | --- | --- | --- |
| Adverse event (MedDRA preferred term) | No. | % | Lower limit (%) | Upper limit (%) |
| Total number of patients (safety population) | 52 | 100.0 | - | - |
| Number of patients with at least one AE of CTCAE grade 3-5 | 52 | 100.0 | 93.2 | 100.0 |
| Mucosal inflammation | 22 | 42.3 | 28.7 | 56.8 |
| Sepsis* / septic shock | 16 | 30.8 | 18.7 | 45.1 |
| Nausea | 9 | 17.3 | 8.2 | 30.3 |
| Pneumonia / atypical pneumonia | 9 | 17.3 | 8.2 | 30.3 |
| Blood bilirubin increased** | 6 | 11.5 | 4.4 | 23.4 |

* including neutropenic, pulmonary, Escherichia, pseudomonal, bacterial and urosepsis

** According to the clinical trial protocol, laboratory parameters outside the normal range were recorded as adverse events only if judged by the investigator to be clinically significant.

*** only AEs listed which occurred in at least 10% of the patients
